# The PANoptotic mosaic of rheumatoid arthritis: epitranscriptomic regulation, systemic relays, and precision death-mode editing

**DOI:** 10.3389/fimmu.2026.1785209

**Published:** 2026-02-26

**Authors:** Shu Li, Lei Wan, Xiaojun Zhang

**Affiliations:** 1 The First Affiliated Hospital of Anhui University of Chinese Medicine, Hefei, China; 2 Anhui University of Chinese Medicine, Hefei, China

**Keywords:** death-mode editing, m6A epitranscriptomics, PANoptosis, rheumatoid arthritis, synovial microenvironment remodeling

## Abstract

The persistence of difficult-to-treat rheumatoid arthritis (D2T-RA) underscores a fundamental disruption in synovial cell death homeostasis, transcending the limitations of conventional cytokine blockade. By integrating multi-omics, molecular imaging, and bio-responsive nanotechnologies, we characterized the PANoptosis framework—a synergistic programmed cell death (PCD) system converging apoptosis, pyroptosis, and necroptosis. Our findings reveal that environmental stressors perturb cellular antioxidant defenses, thereby precipitating PANoptosome assembly through mechanisms such as autoantibody-mediated biophysical triggers. Systemic crosstalk, spanning lung-derived inflammatory signals and gut metabolic rheostats, orchestrates synovial fate. Mechanistically, epitranscriptomic RNA methylation and dysregulated molecular switches within the PANoptosome drive inflammatory flares, while distal effects involve extracellular vesicle-mediated cartilage damage. Therapeutic interventions, such as bio-responsive nanoplatforms, effectively reprogram death modes toward inflammatory resolution. We conclude that PANoptosis is a central driver of RA pathogenesis, and its precision targeting via “death-mode editing” represents a paradigm shift from broad immunosuppression toward curative interventions. This work establishes a comprehensive PANoptic model and identifies actionable therapeutic avenues, offering transformative potential for the clinical management of RA.

## 1 Introduction

For decades, the therapeutic landscape of rheumatoid arthritis (RA) has been dominated by the dual pillars of cytokine blockade and systemic immunosuppression (1–3). Despite these advancements, “difficult-to-treat” RA (D2T-RA) persists as a formidable clinical challenge. In these patients, the synovial pannus remains a pseudo-malignant, invasive tissue even when systemic inflammatory markers have been successfully attenuated (4, 5). This clinical paradox suggests that RA is not merely a disease of immune cell infiltration, but rather a fundamental collapse of cellular death homeostasis within the arthritogenic synovial niche (6). We propose that the synovial microenvironment is governed by a PANoptic framework—an integrated system of regulated cell death (RCD) that transcends the conventional boundaries of apoptosis, pyroptosis, and necroptosis (7, 8). Central to this framework is the PANoptosome, a multi-protein molecular scaffold that serves as a critical integration platform for environmental and inflammatory stressors (9). Recent evidence further underscores the role of selective kinase inhibitors, such as Janus kinase 1 (JAK1), in fine-tuning these interconnected death pathways (10). Within the RA joint, the molecular dysregulation of PANoptosome components—exemplified by the Caspase-8/GSDME switch—converts homeostatic resolution signals into chronic pro-inflammatory flares (11, 12). This Review delineates the “synovial mosaic” of RA, characterizing how intra-articular death signatures are sculpted by environmental priming through the Keap1-Nrf2 axis (13, 14) and maintained by an epitranscriptomic regulatory layer of RNA methylation (15). Furthermore, we extend the scope of RA pathogenesis beyond the synovial lining to the neuro-immune-death axis, where a failure of the efferocytic machinery in the dorsal root ganglia (DRG) dictates the transition from acute inflammation to persistent pain (16, 17). Crucially, we highlight how advancements in molecular imaging and bio-responsive nanoplatforms are facilitating a transition from broad immunosuppression toward the pharmacological “editing” of death modes, aiming for the targeted resolution of the disease-driving microenvironment (18, 19). Finally, we explore the “gut–joint PANoptic relay,” where dysbiosis-derived metabolites and short-chain fatty acids (SCFAs) function as systemic rheostats that gate the susceptibility of synovial cells to inflammatory demise (20).

## 2 The systemic mosaic: multi-organ relays in RA

### 2.1 Environmental priming and the lung–joint axis

The initiation of rheumatoid arthritis (RA) represents a silent prologue scripted by the exposome (14). Environmental “first hits” recalibrate cellular thresholds for inflammatory cell death, priming the transition from health to autoimmunity. Proteomic profiling has identified lysozyme (LYZ) as a pivotal orchestrator that, when overexpressed in early RA macrophages, exacerbates TNF-induced inflammatory death (21). Furthermore, chronic exposure to particulate matter precipitates the collapse of the Keap1-Nrf2 antioxidant axis, impairing cellular resistance to oxidative stress (13, 22).

A central enigma in RA pathogenesis is the translation of pulmonary insult into synovial destruction. We propose a systemic relay model—the Lung–Joint Axis—where lung-derived neutrophil extracellular traps (NETs) and exosomes facilitate the delivery of citrullinated damage-associated molecular patterns (DAMPs) to the joint (23, 24). Viral pathogens, notably Parvovirus B19, function as biological initiators; the B19-NS1 protein activates NRF2-mediated stress responses and mTOR signaling, recapitulating the synovial niche even in the absence of conventional autoantibodies (25). From a systems perspective, shared WNT/STAT3 signaling pathways within this axis may explain the epidemiological link between RA-associated interstitial lung disease and increased carcinogenesis risk (26). Additionally, senescent cells adopting a senescence-associated secretory phenotype (SASP) act as chronic inflammatory reservoirs that sustain this pathogenic state (27, 28). To interrupt this systemic relay, autophagy inhibitors have demonstrated high precision in blocking pathogenic NET release without compromising systemic host defense (24), offering a targeted strategy to decouple pulmonary triggers from synovial inflammation.

### 2.2 The gut-joint metabolic rheostat

Gut dysbiosis in rheumatoid arthritis (RA) facilitates the systemic translocation of pro-inflammatory metabolites, effectively bridging intestinal imbalance with synovial inflammation. Functional metabolomics has identified palmitic acid (PA)—often elevated in the context of high-fat diets and dysbiosis—as a potent systemic trigger of synovial pathology (29). PA exacerbates the expression of pro-inflammatory mediators by activating the NLRP3/Caspase-1/GSDMD-N pyroptotic axis in fibroblast-like synoviocytes (FLSs), thereby “priming” the arthritogenic niche for clinical flares (29).

Conversely, commensal-derived metabolites function as endogenous inhibitors of the PANoptosome. Short-chain fatty acids (SCFAs), such as butyrate, modulate T-lymphocyte homeostasis by inducing apoptosis in activated T cells through histone deacetylase (HDAC) inhibition and Fas upregulation (20). This mechanism effectively prevents the intra-articular accumulation of senescent, senescence-associated secretory phenotype (SASP)-producing T cells. Furthermore, dietary polyphenols such as anthocyanins (e.g., PSPA) have been shown to remodel the gut microbiota by enriching beneficial taxa like *Akkermansia* and *Lactobacillus*. This microbial shift correlates with the attenuation of synovial pyroptosis and the restoration of PI3K/AKT signaling (30). A landmark study in 2025 identified Urolithin A (UA), a gut-derived metabolite of natural polyphenols, as a critical “molecular rheostat” in death-mode regulation. UA attenuates RA pathogenesis by suppressing the NF-κB pathway and concurrently activating AMPK signaling, thereby inhibiting GSDMD-mediated pyroptosis in synovial fibroblasts (31).

### 2.3 The neuro-efferocytic gap and persistent pain

A transformative paradigm shift emerged in 2025 with the discovery that chronic arthritis pain—which often persists independently of local synovial disease activity—is underpinned by a failure of the death-resolution machinery within the dorsal root ganglia (DRG) (16). The neuro-immune interface further exerts direct regulatory control over synovial death modes via the cholinergic anti-inflammatory pathway. Specifically, the α7 nicotinic acetylcholine receptor (α7nAChR) has emerged as a pivotal checkpoint inhibiting the pyroptosis of RA synovial fibroblasts. By suppressing the NLRP3/GSDMD axis, α7nAChR activation effectively “reprograms” synoviocyte fate, thereby mitigating inflammatory flares and subsequent tissue destruction (32). Crucially, this operates as a bidirectional loop: the failure of efferocytosis in the DRG leads to the persistent release of neuropeptides (e.g., Substance P, CGRP), which are retrogradely transported to the joint to promote synovial vasodilation and maintain the inflammatory ‘fire’ even after local cytokines are suppressed. Pharmacological modulation of this axis, such as the inhibition of BDNF/TrkB signaling via ANA-12, significantly attenuates glial activation and pro-inflammatory cytokine secretion (17). Furthermore, reactive oxygen species (ROS)-scavenging hydrogen nanotherapy represents a dual-action approach, simultaneously alleviating synovial inflammation and central pain sensitization (33).

### 2.4 Synovial redox gating: Keap1-Nrf2 axis

Investigation into Salvianolic acid B demonstrates that restoring the Keap1-Nrf2 axis via direct binding to the Keap1-Arg415 residue suppresses pyroptosis, identifying this residue as a pivotal gatekeeper of synovial death homeostasis (13). Furthermore, the systemic ramifications of redox collapse extend to extra-articular complications. Recent evidence indicates that silymarin, a natural flavonoid, attenuates cardiac injury and inflammation in rats with adjuvant-induced arthritis (AIA). This cardioprotective effect is mediated by the Nrf2/SLC7A11/GPX4 axis, which suppresses ferroptosis and inflammatory responses in cardiac tissues, suggesting that Nrf2-regulated death pathways are essential for managing RA as a multi-organ systemic pathology (34).

### 2.5 Senescent reservoirs

Environmental stressors accelerate the acquisition of a senescence-associated secretory phenotype (SASP) in immune cells, particularly CD4^+^ and CD8^+^ T cells (27). Functioning as intra-articular “inflammatory reservoirs,” these cells secrete TNF-α and IL-6, which provide the requisite “second hit” for the persistent assembly of synovial PANoptosomes (27, 28). This transition is molecularly governed by CCNE2; the inhibition of CCNE2 effectively triggers senescence-associated apoptosis in synoviocytes, representing a promising senolytic strategy (28). Furthermore, the epitranscriptomic regulation of cell fate is intrinsically coupled to the crosstalk between non-coding RNAs (ncRNAs) and programmed cell death (PCD) pathways (35). Dysregulated long non-coding RNAs (lncRNAs) and circular RNAs (circRNAs) within the RA synovial niche serve as master orchestrators, modulating the sensitivity of fibroblast-like synoviocytes (FLSs) to apoptotic and pyroptotic stimuli, thereby driving the progression from acute inflammation to chronic structural damage. This systemic failure of death-mode resolution across diverse anatomical sites—constituting the “synovial mosaic” of RA—is systematically summarized in **Table 1**.

**Table 1:** The synovial mosaic: multi-organ death and resolution signatures.

Anatomical site	Primary cell population	Predominant signature	Key molecular driver	Impact on RA pathology	Ref.
Dorsal Root Ganglia	Macrophages	Defective Efferocytosis	MerTK/12/15-LOX	Drives persistent, nociplastic pain.	(16)
Gut Niche	Microbiota/T cells	SCFA-mediated Apoptosis	HDAC/Fas pathway and SCFAs	Systemic gating of T-cell senescence.	(20, 30)
Articular Cartilage	Chondrocytes	Remote Apoptosis	miRNA-15/CIAPIN1	Irreversible cartilage loss.	(36)
Synovial Lining	RA-FLSs	PANoptotic Escape	USP5/FTO/METTL3	Invasive pannus formation.	(15, 37, 38)
Bone Niche	Osteoclasts	Longevity-Gating	AMPK/HIF-1α	Marginal bone erosions.	(39, 40)

**Figure 1:**
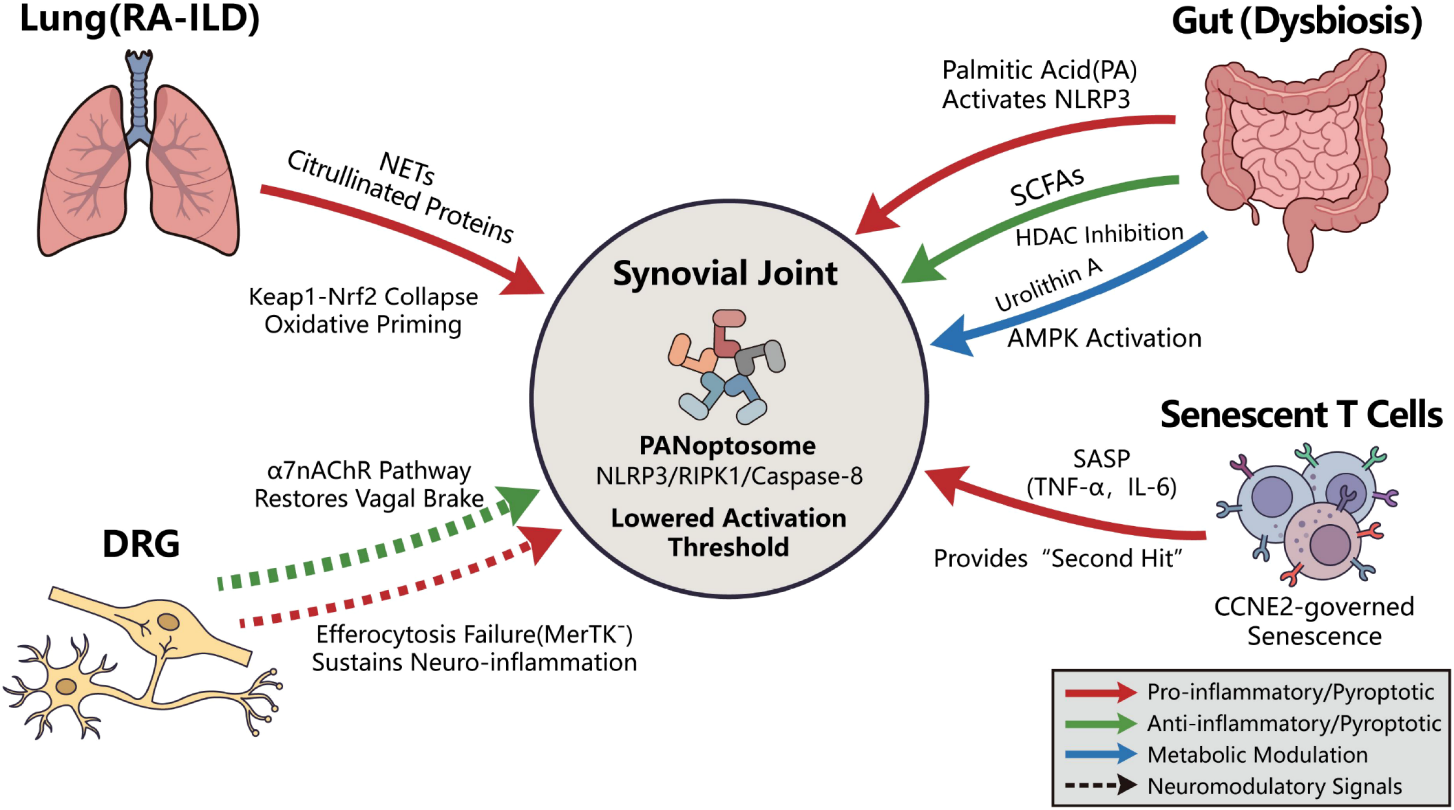
The systemic mosaic: multi-organ relays priming the synovial niche. This schematic illustrates how signals from distant organs converge on the joint to lower the threshold for inflammatory cell death (PANoptosis). **Lung–Joint Axis (Top Left)**: In RA-ILD, lung-derived NETs and citrullinated proteins disrupt the systemic antioxidant defense (Keap1–Nrf2), causing “oxidative priming” of synovial cells. **Gut–Joint Axis (Top Right):** A metabolic dichotomy exists: pathogenic Palmitic Acid (PA) fuels inflammation (NLRP3 activation), while beneficial metabolites like SCFAs and Urolithin A act as “brakes” via AMPK activation. **Senescent Reservoirs (Bottom Right):** Aged T cells (CD4^+^/CD8^+^) secrete a cocktail of inflammatory cytokines (SASP: TNF-α, IL-6), providing the critical “second hit” needed to trigger cell death complexes. **Neuro–Joint Axis (Bottom Left):** A failure in neural regulation occurs due to the loss of the “vagal brake” (α7nAChR) and defective debris clearance (MerTK dysfunction) in the dorsal root ganglia (DRG). **Synovial Convergence (Center):** These systemic triggers cumulatively destabilize the synovial niche, converting environmental stress into explosive PANoptotic death.

In summary, these multi-organ relays do not operate in isolation; rather, they function as critical upstream priming signals (**Figure 1**). Whether via gut metabolite-induced NLRP3 activation or lung-derived damage-associated molecular pattern (DAMP)-mediated ZBP1 sensing, these systemic inputs lower the biophysical threshold for PANoptosome assembly. Consequently, the synovial niche serves as a molecular crucible where environmental “hits” are translated into the programmed execution of PANoptosis, a process ultimately governed by an underlying epitranscriptomic script.

## 3 The molecular machinery of synovial PANoptosis

**Figure 2:**
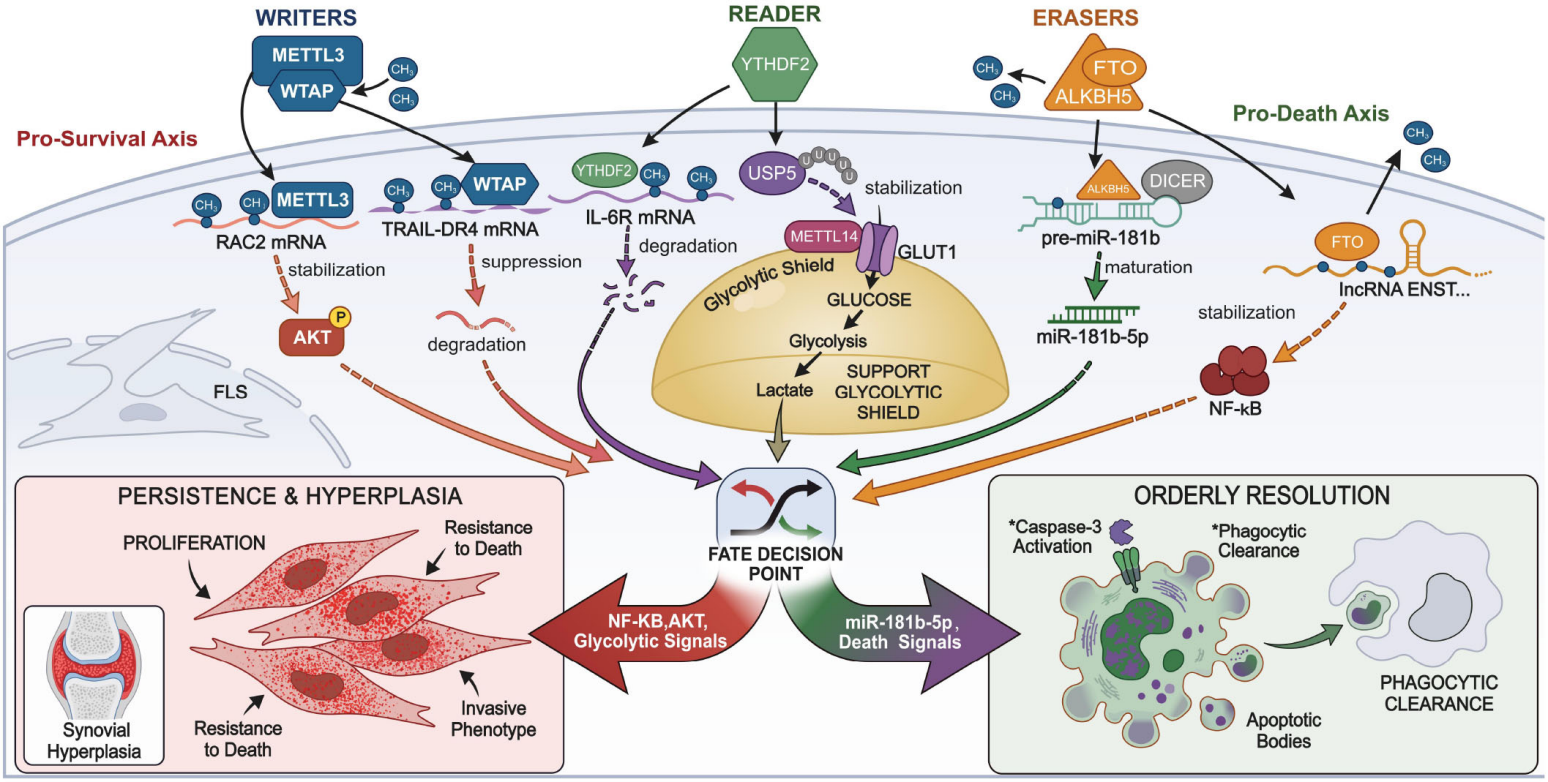
The “software” layer: epitranscriptomic and metabolic gating of synovial cell fate. This diagram illustrates how chemical modifications on RNA (m6A) and metabolic signals act as a “software” code to decide cell survival. **Pro-survival Axis:** The Eraser FTO and Writers (METTL3, WTAP) stabilize key survival genes (e.g., RAC2, lncRNAs), activating NF-κB/AKT pathways to drive synovial hyperplasia. **Pro-resolution Axis:** Conversely, the Eraser ALKBH5 facilitates miR-181b maturation, while the Reader YTHDF2 degrades IL-6R mRNA, promoting orderly cell death (resolution). **The Metabolic Shield:** Centrally, the USP5-METTL14-GLUT1 axis links glucose metabolism to m6A modification, maintaining a “glycolytic shield” that protects aggressive cells from dying.

**Figure 3:**
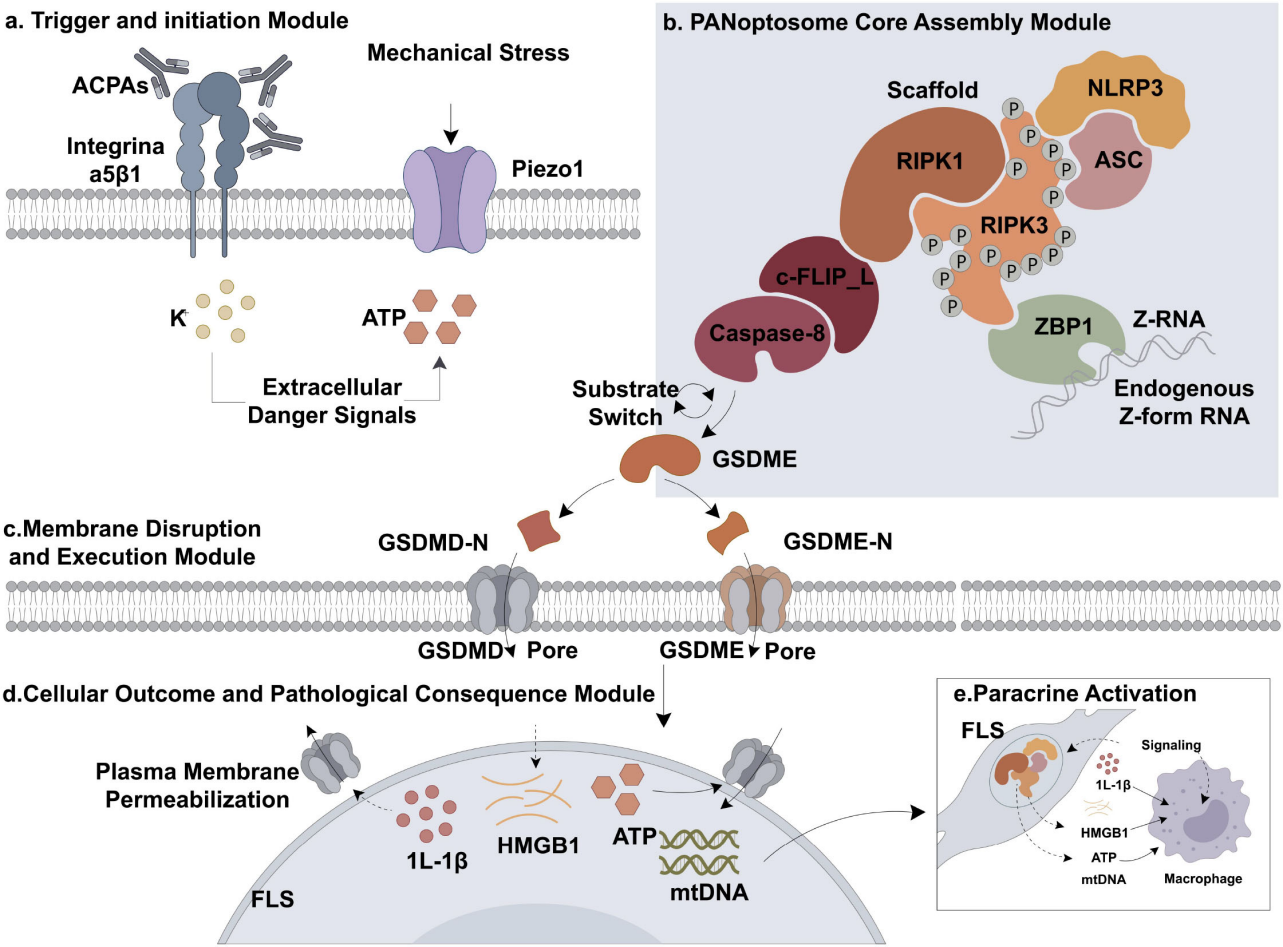
The “hardware” scaffold: PANoptosome assembly and execution in RA. This schematic details the physical machinery (“Hardware”) that executes cell death in the joint. **Step A:** Priming Triggers: Environmental stresses—such as antibodies (ACPA), mechanical pressure (Piezo1), or ionic imbalances—act as “first hits” to alert the cell. **Step B:** The Core Assembly: These signals trigger the formation of the PANoptosome, a multi-protein complex containing ZBP1, RIPK1/3, and NLRP3. **Step C:** The Fatal Switch: A critical miswiring event occurs where Caspase-8 switches substrates. Instead of causing silent apoptosis, it cleaves GSDME, leading to explosive cell rupture (pyroptosis) and massive release of inflammatory signals (DAMPs/IL-1β).

The molecular landscape of synovial PANoptosis is organized into two primary layers: a regulatory ‘Software’ layer governed by epitranscriptomic modifications (**Table 2**) (**Figure 2**) and a structural ‘Hardware’ scaffold composed of multi-protein complexes (**Table 3**) (**Figure 3**).

**Table 2:** The software layer: epitranscriptomic and metabolic gating of cell death.

Regulator type	Specific molecule	Mechanism of action	Primary target/axis	Impact on synovial niche	Ref.
Eraser	FTO	m6A Demethylation	lncRNA ENST00000619282	Stabilizes anti-apoptotic lncRNAs; locks FLSs in a survival state.	(15)
Eraser	ALKBH5	m6A Demethylation	pre-miR-181b-1	Facilitates miR-181b maturation to promote FLS apoptosis.	(41)
Writer	METTL3	m6A Methylation	RAC2 mRNA/AKT axis	Increases mRNA stability; enhances motility and ROS resistance.	(37)
Writer	WTAP	m6A Methylation	TRAIL-DR4	Reduces sensitivity to death ligands; drives synovial hyperplasia.	(42)
Reader	YTHDF2	mRNA Degradation	IL-6R mRNA	Tunes cellular sensitivity to IL-6-driven pro-survival signaling.	(43)
Crosstalk	USP5	Deubiquitination	METTL14/GLUT1	Stabilizes the writer complex to maintain the “glycolytic shield.”	(38)

**Table 3:** The PANoptic hardware: core molecular and biophysical components in RA.

Component	Canonical role	Pathogenic miswiring in RA	Consequence	Ref.
Caspase-8	Apoptosis Initiator	Switched to cleave GSDME in RA-FLSs.	Converts resolution to pyroptosis.	(11, 44)
RIPK1/3	Necroptosis Execution	Hyper-phosphorylated; facilitates NLRP3 assembly.	Promotes necro-pyroptotic synergism.	(9, 45, 46)
NLRP3	Inflammasome Sensor	Lowered threshold due to Keap1-Nrf2 failure.	Drives synovial inflammatory flares.	(13, 47)
GSDMD/E	Pore Formation	Increased membrane translocation and cytolysis.	Massive release of DAMPs and IL-1β.	(11, 12, 31)
ZBP1	Nucleic Acid Sensor	Hyper-activated by mtDNA/cGAS-STING axis.	Triggers sterile inflammation via endogenous Z-DNA/mtDNA sensing	(25, 48, 49)
Piezo1	Mechanosensor	Over-activated by synovial fluid pressure.	Translates mechanical stress into NLRP3 assembly.	(50)

### 3.1 The “software” layer: m6A-mediated gating

#### 3.1.1 The epitranscriptomic checkpoint: software control of cell fate

Cellular decision-making is governed by an epitranscriptomic checkpoint (8, 15). The eraser FTO stabilizes anti-apoptotic lncRNA ENST00000619282, activating NF-κB to lock FLSs in a survival state (15). Conversely, ALKBH5 demethylates pre-miR-181b-1 to facilitate miR-181b-5p maturation and promote apoptosis (41). Writers like METTL3 and WTAP architect the aggressive phenotype by stabilizing RAC2 and TRAIL-DR4 (37, 42). The reader YTHDF2 acts as an inflammatory rheostat for IL-6R stability (43). Metabolic signals are integrated via the USP5/METTL14 crosstalk, maintaining a “glycolytic shield” against PANoptic bursts (38, 51).

#### 3.1.2 Erasers as gatekeepers of death escape

The shift from homeostatic apoptosis to pathological persistence is often mediated by m6A “erasers.” The demethylase FTO has been identified as a critical driver of apoptosis escape in FLSs. By reducing m6A levels on the lncRNA ENST00000619282, FTO enhances its stability, which in turn activates NF-κB signaling—effectively locking the synoviocyte in a pro-inflammatory survival state (15). Conversely, the eraser ALKBH5 appears to function as a resolution-promoter; its demethylation of pre-miR-181b-1 facilitates the maturation of miR-181b-5p, which sensitizes FLSs to apoptotic triggers (41). Furthermore, the IL-21/IL-21R axis has been found to synergistically promote FLS survival during endoplasmic reticulum stress (ERS) by modulating the autophagy-ERS balance via USP18 (52).

#### 3.1.3 Writers as phenotypic architects

The m6A “writer” complex, including METTL3 and WTAP, functions to architect the aggressive synovial phenotype. METTL3 stabilizes the transcripts of RAC2, thereby activating AKT signaling and enhancing FLS motility and death-resistance (37). Similarly, WTAP suppresses the extrinsic apoptotic pathway by mediating the m6A methylation of TRAIL-DR4 mRNA, reducing its expression (42).

#### 3.1.4 Readers and metabolic rheostats

The final execution relies on “readers” like YTHDF2, which governs the degradation of the IL-6R transcript, acting as an inflammatory rheostat (43). This layer integrates metabolic signals through the USP5/METTL14 crosstalk, where deubiquitination maintains a “glycolytic shield” that prevents PANoptic bursts (38, 51). This metabolic gating is now known to involve refined scripts for alternative substrates. PCK1 has been identified as a pivotal hub gene that distinguishes the lactate metabolism of RA from OA; its knockdown effectively disrupts the metabolic resilience of RA-FLS and triggers apoptosis (53). In parallel, CD36-mediated fatty acid metabolic reprogramming acts as another survival shield. Upregulation of CD36 in RA-FLS activates the PI3K/AKT/mTOR signaling axis, which enhances mitochondrial resistance and suppresses homeostatic cell death, driving the invasive FLS phenotype (54). A landmark 2025 meta-analysis confirms that the PI3K/AKT/mTOR axis is the robust translational marker for drug response in RA death-mode editing (55).

It is important to note that the “glycolytic shield” hypothesis and associated m6A-metabolic crosstalk are primarily supported by *in vitro* cell line studies and murine CIA models (38, 51). Direct validation in human RA synovial tissue using spatial metabolomics and patient-derived primary cells remains a critical translational gap. Species-specific differences in metabolic flux and PANoptosome assembly thresholds warrant cautious interpretation pending rigorous clinical validation.

### 3.2 The “hardware” scaffold: PANoptosome structure

#### 3.2.1 Triggering and assembly

Biophysical triggers for PANoptosome activation are provided by anti-citrullinated protein antibodies (ACPAs). By binding to integrin α5β1, ACPAs recruit SFK kinase to induce ATP release and K^+^ efflux via the TWIK2 channel (12). This creates the specific ionic imbalance required for NLRP3-dependent PANoptosome assembly (12, 47, 48). This biophysical threshold is further lowered by the Piezo1 mechanosensitive channel, which translates synovial fluid pressure into NLRP3 inflammasome assembly (50). Calcium signaling serves as the universal secondary messenger for these decisions (56), with the mitochondrial calcium uniporter (MCU) specifically dictating the metabolic reprogramming and invasiveness of death-resistant FLSs (57).

#### 3.2.2 The PANoptosome hub: a molecular diverter of cell fate

The PANoptosome dictates whether a cell undergoes homeostatic turnover or inflammatory explosion (8, 9) (**Table 1**). In aggressive RA-FLSs, Caspase-8 is miswired to cleave GSDME instead of executing silent apoptosis, switching resolution into a pyroptotic flare (11). In this death-mode decision crucible, ferroptosis—an iron-dependent form of RCD—acts as a lateral amplifier of synovial destruction (58). RIPK3 acts as the choreographer of this synergism between necroptosis and NLRP3 assembly (9, 45).

#### 3.2.3 Necro-pyroptotic synergism and RIPK3

The boundary between necroptosis and pyroptosis is functionally blurred. RIPK3 facilitates NLRP3 assembly and GSDMD-mediated pore formation (9, 45). This synergy ensures that even if one death pathway is blocked, the cell remains committed to an inflammatory demise via the necrosome—a feedback loop that can be disrupted by targeting the RIPK3-NLRP3 axis (45, 46). Crucially, the assembly of the ZBP1-PANoptosome in RA is also triggered by the failure of self-RNA homeostasis. Under synovial hypoxia, the compromised expression of ADAR1 leads to the accumulation of endogenous Z-form RNA, acting as high-affinity ‘self-ligands’ for ZBP1.

### 3.3 The switch: Caspase-8 and the divergence of cell fate

Beyond its canonical role as the initiator of extrinsic apoptosis, Caspase-8 functions as the catalytic and structural rheostat of the PANoptosome in RA. In RA-FLSs, the overactivation of IAPs and the sequestration of Caspase-3 create a molecular bottleneck. Consequently, Caspase-8 undergoes a substrate-preference shift, engaging GSDME at the Asp270 site. This ‘backup plan’ transforms a suppressed apoptotic signal into an explosive pyroptotic flare. Recent evidence (49) reveals that Caspase-8 does not solely act as a protease but also as a scaffold for NLRP3 assembly. In RA-FLSs, Caspase-8 is redirected by c-FLIP_L, which forms a heterodimer that preferentially activates the ZBP1-RIPK3 axis, mirroring the molecular switch observed in pathogen-induced PANoptosis, switching the cell from a “silent” apoptotic exit to an explosive pyroptotic program (59). The pathogenic reach of this switch extends to rheumatoid sarcopenia. Recent evidence (44) underscores that TNF-α drives muscle atrophy by hijacking the Caspase-8/Caspase-3/GSDME-mediated pyroptotic axis. When primed by chronic TNF-α, Caspase-8 activation facilitates GSDME cleavage, triggering a lytic death that exacerbates tissue loss. This reinforces the necessity of targeting the Caspase-8 switch not only to preserve joint integrity but also to mitigate the systemic burden of the disease.

## 4 Downstream consequences: tissue demise and erosive frontier

### 4.1 The remote PANoptic relays

Joint destruction involves remote PANoptic relays. A failure of MerTK-mediated efferocytosis of apoptotic neutrophils in the DRG drives persistent mechanical hypersensitivity (16, 17). Remote chondrocyte demise is triggered by FLS-derived sEVs carrying miRNA-15–29148 targeting CIAPIN1 (36, 60). Subchondral bone survival is gated by the AMPK/HIF-1α axis in the hypoxic niche (39, 40). Pathological B-cell aggregates maintain the DAMP pool, which can be remodeled using bioactive glass IDPs (61).

### 4.2 Far-field toxins and chondrocyte demise

The destruction of articular cartilage is increasingly recognized as a “remote PANoptic relay.” FLS-derived small extracellular vesicles (sEVs) act as long-range commands, transporting miRNA-15–29148 across the joint space (36). Once internalized by chondrocytes, this miRNA targets CIAPIN1 (Cytokine-Induced Apoptosis Inhibitor 1), effectively disabling the chondrocyte’s internal survival rheostat and triggering premature apoptosis even in paucimmune states (36, 60). This remote killing is amplified by the CXCL10/CXCR3 axis, which recruits further inflammatory precursors to the cartilage-pannus junction, creating a self-amplifying cycle of matrix degradation (60).

### 4.3 Osteoclast metabolic gating and the B-cell niche

In the marginal zones of bone erosion, the survival of bone-resorbing osteoclasts is governed by a hypoxia-death checkpoint. The AMPK/HIF-1α axis prevents the PANoptic demise of osteoclast precursors in the hypoxic RA niche, converting synovial metabolic stress into a persistent bone-resorption signal (39, 40). This environment is further stabilized by pathological B-cell aggregates that maintain a localized DAMP pool. Recent evidence suggests that targeted B-cell depletion—utilizing bioactive glass ionic dissolution products (IDPs)—can remodel this osteoimmunological microenvironment, triggering osteoclast apoptosis and shifting the balance toward osteoblastic bone repair (61). Advanced nanozyme coatings can now reprogram this niche by converting H_2_O_2_ into O_2_, thereby preventing macrophage apoptosis and shifting polarization toward the M2 phenotype. Classical agents like iguratimod also exert therapeutic effects by modulating this AMPK/HIF-1α axis to suppress osteoclast differentiation (39).

In summary, PANoptosis in RA represents a ‘messy death’ that fundamentally sabotages the resolution of inflammation. Unlike homeostatic apoptosis, which is ‘silent’ and facilitates clearance, the lytic nature of PANoptotic FLSs releases a massive pool of DAMPs (e.g., HMGB1) that overwhelms the efferocytic capacity of local MerTK+ macrophages. This combination of explosive cell death and ‘cleaning failure’ (impaired efferocytosis) creates a feed-forward loop that perpetuates the autoimmune response within the synovial mosaic.

### 4.4 Subtype-specific integration of systemic triggers

Collectively, these multi-organ relays converge on the synovial niche to prime PANoptosome assembly. A critical unresolved question is whether RA subtypes differentially engage these pathways.

Emerging evidence suggests distinct “trigger fingerprints” across RA subtypes:

- Seropositive RA (ACPA+/RF+) preferentially activates the lung–joint axis. ACPA-driven ionic flux (integrin α5β1/TWIK2/NLRP3) (12) sustains PANoptosome assembly, amplified by citrullinated DAMPs and neutrophil extracellular traps from airway inflammation (23, 24). This dual priming may explain the erosive phenotype and treatment resistance of seropositive disease.
- Seronegative RA, lacking ACPA-mediated triggering, appears more reliant on the gut–joint metabolic relay (palmitic acid/NLRP3) (29), mechanical stress (Piezo1) (50), and viral PAMPs (B19-NS1/ZBP1) (25), yielding distinct PANoptotic kinetics.
- Validation gap: Systematic profiling of PANoptosome components (RIPK1/3, ZBP1, NLRP3, GSDMD) across synovial compartments, stratified by autoantibody status and relay activation markers (citrullinated peptides, microbiome metabolites), is absent. Single-cell spatial transcriptomics and multiplexed imaging (CODEX, IMC) are essential.

Therapeutic implication: Subtype-tailored strategies may be required—ACPA blockade plus NLRP3 inhibition for seropositive disease versus microbiome modulation or Piezo1 antagonism for seronegative cases.

## 5 Therapeutic reprogramming: editing the death script

We propose “Death-Mode Editing” as a holistic paradigm shift in RA therapy. This approach moves beyond broad cytokine suppression toward the precise visualization, redirection, and eradication of the arthritogenic niche. The integration of [^11^C]CMP1 PET mapping of RIPK1 allows clinicians to visualize necroinflammatory “hot-spots” *in vivo* (8, 18). Precision execution then utilizes bio-responsive platforms—such as GelMA microneedles to redirect Caspase-8 (11, 13, 27, 62) or CLT-FELipos to induce selective ER-stress apoptosis via KDEL targeting (38, 63)—to eliminate aggressive FLSs while restoring immune homeostasis through engineered efferocytic vesicles (43, 54, 62). Furthermore, epitranscriptomic “software” layers, such as the FTO/m6A axis, can be targeted by Xinfeng Capsule (XFC) to reverse apoptosis escape (5, 15).

### 5.1 Spatiotemporal navigation: molecular imaging

A primary obstacle in RA management is the lack of tools to visualize the intra-articular death landscape. A 2025 breakthrough identifies [^11^C]CMP1 PET as a high-affinity radiotracer for RIPK1, enabling the first-ever spatial mapping of necroinflammatory “hot-spots” *in vivo* a technique adapted from RIPK1 neuroimaging (8, 18). This “molecular compass” allows clinicians to identify patients with active PANoptic flares, facilitating the selection of individuals for targeted death-mode interventions rather than broad-spectrum biological therapy (8, 18). Critically, RIPK1+ synovial burden may differ between subtypes: seropositive patients with chronic lung–joint axis activation (ACPA/ionic flux) (12, 23, 24) may exhibit sustained, diffuse signals, whereas seronegative cases reliant on intermittent triggers (gut-metabolic/mechanical) (29, 50) may show spatially restricted patterns. Longitudinal [^11^C]CMP1 PET studies correlating uptake with autoantibody titers and systemic relay biomarkers are urgently needed to validate the subtype-specific models proposed in Section 4.4. Additionally, circulating AIM2 methylation levels have emerged as a novel epigenetic biomarker to predict therapeutic response in difficult-to-treat (D2T) RA (9, 64), while near-infrared fluorescent probes (e.g., BHD) enable real-time monitoring of microenvironmental alterations associated with synovial demise (15). Integrating [^11^C]CMP1 PET with circulating m6A signatures (9) and relay-specific markers would operationalize the three-tier stratification algorithm detailed in Section 5.4, ensuring the right therapy reaches the right patient phenotype.

### 5.2 Bio-responsive execution: multi-dimensional editing

While JAK inhibitors act as inadvertent top-down ‘PANoptotic silencers’ by downregulating ZBP1 and RIPK1, future strategies aim for direct enzymatic and metabolic “editing” of the death script.

#### 5.2.1 Molecular execution platforms

Redirecting Caspases: Precision pyroptosis can be executed via the VIP-modified ‘D.ZAN’ platform, which utilizes ultrasound-triggered Zn²^+^ release to activate the Caspase-1/GSDMD axis specifically in hyper-activated FLSs (19, 65, 66). Similarly, GelMA-SilMA hydrogel microneedles inhibit Caspase-8 miswiring, switching the cellular trajectory from lytic pyroptosis back toward silent apoptosis (13, 27, 62).

Neuro-Immune Checkpoints: The α7 nicotinic acetylcholine receptor (α7nAChR) has emerged as a key checkpoint; its activation suppresses the NLRP3/GSDMD pathway in RA-FLSs, effectively “editing” the synoviocyte fate to prevent inflammatory flares (32, 67).

Organelle-Targeted Lethality: Enzyme-responsive CLT-FELipos induce ER stress-mediated apoptosis specifically in FAPα^+^ FLSs (63). In parallel, PPy-FePi-MTX nanoparticles achieve synergistic anti-inflammatory effects by blocking cytoprotective autophagy to enhance apoptosis/ferroptosis in M1 macrophages (27, 45).

New Delivery Platforms: Melittin-chondroitin sulfate cryo-microneedles allow for targeted induction of apoptosis in synovial fibroblasts (68, 69). To address the ‘software’ layer, self-assembled Sinomenine-Glycyrrhizic acid nanohydrogels reverse neutrophil apoptosis delay via NF-κB pathways (70), while Cold Atmospheric Plasma (CAP) selectively induces apoptosis through redox editing (71, 72).

#### 5.2.2 Overcoming resistance: breaking the metabolic and software shields

Aggressive FLSs maintain a “glycolytic shield” and metabolic resilience. Inhibiting the USP5/METTL14 axis (6, 38) or targeting PCK1-mediated lactate metabolism (53, 54) disrupts this resilience. Furthermore, CD36-mediated fatty acid reprogramming activates the PI3K/AKT/mTOR survival axis (32, 54), a pathway that integrates with RNF19A-mediated drug resistance (53, 73).

#### 5.2.3 Specific pharmacological scripts provide re-sensitization

Auranofin covalently binds PRDX1/2 to trigger ROS-dependent cell death (66, 67). Kaempferol restores the Bax/Bcl-2 balance and induces mitochondrial apoptosis via inhibition of the PI3K/Akt pathway (74, 75). Cannabigerol (CBG) neutralizes necro-pyroptotic synergism by downregulating NLRP3 and NF-κB (45, 75, 76). Dapagliflozin further targets the crosstalk between apoptosis and autophagy via AMPK activation (69). These targeted pharmacological scripts provide a sophisticated toolkit for re-sensitizing resistant synoviocytes to homeostatic demise. A comprehensive list of candidate agents, including natural monomers, TCM formulations, and repurposed clinical drugs, along with their specific molecular targets and PANoptic gating mechanisms, is systematically summarized in **Table 4**.

**Table 4:** Pharmacological scripts: precision death-mode modulators and repurposed drugs.

Candidate agent	Biological source/class	Primary molecular target	PANoptic gating mechanism	Ref.
Salvianolic acid B	Salvia miltiorrhiza	Keap1 (Arg415)	Disrupts Keap1-Nrf2 interaction; inhibits ROS-induced pyroptosis	(13)
Xinfeng Capsule (XFC)	TCM Formulation	FTO (m6A Demethylase)	Inhibits FTO to stabilize m6A-modified lncRNAs; reverses FLS apoptosis escape	(15)
ANA-12	TrkB Antagonist	BDNF/TrkB	Reduces DRG glial activation to resolve persistent pain	(17)
Silymarin	Flavonoid	Nrf2/SLC7A11	Suppresses cardiac ferroptosis	(34)
Kaempferol	Flavonoid	PI3K/Akt path	Reverses FLS apoptosis resistance	(74)
Cannabigerol	Cannabinoid	TLRs/NLRP3	Blocks necro-pyroptotic synergism	(75)
Auranofin	Gold(I) Complex	TrxR1/PRDX1/2	Triggers ROS-dependent cell death by binding PRDX	(67)
Dapagliflozin	SGLT2 Inhibitor	AMPK/Hedgehog	Targets crosstalk between apoptosis, autophagy, and Hedgehog signaling	(69)
Isorhapontigenin	TCM Monomer	FDPS	Inhibits AKT/ERK pathways to suppress FLS aggression	(77)
Clematichinenoside AR	Clematis chinensis	HIF-1α/VEGFA	Inhibits synovial angiogenesis via death-mode editing	(78)
Songorine (8a)	Diterpenoid alkaloid	NLRP3 (Cys residues)	Covalent inhibition of NLRP3; blocks Caspase-1 and GSDMD cleavage	(72)

### 5.3 Resetting the niche via induced efferocytosis

The transition from “killing” to “niche-resetting” requires restoring efferocytic capacity. Engineered vesicles (D@ApoEVFasL) promote macrophage efferocytosis (62), established as a critical resolution mechanism. This is complemented by systemic rheostats like New Bitongling (NBTL), which suppresses Mapt expression to restore mitochondrial function (66, 74), and pH-responsive hybrid nanoparticles (Pae-PPNPs-DS) that modulate the STAT axis to promote the resolution of inflammation (52, 79). Achieving antigen-specific, drug-free remission remains the ultimate goal of PANoptic niche eradication (80). The diverse array of precision platforms enabling this transition—from spatiotemporal mapping of ‘hotspots’ to the sophisticated bio-responsive editing of death modes—is comprehensively integrated in **Table 5**.

**Table 5:** Integrated precision platforms for PANoptics: from spatiotemporal mapping to death-mode editing.

Platform category	Representative systems	Key trigger/stimulus	Targeted PANoptic component	Diagnostic spatiotemporal navigation	Ref.
Diagnostic & Imaging Probes	[^11^C]CMP1 PET; BHD Fluorescent Probe	RIPK1 binding; Viscosity sensing	RIPK1 expression; Micro-viscosity	Spatiotemporal Navigation: *In vivo* mapping of “necro-inflammatory hotspots”	(15, 18)
Advanced Microneedles (MNs)	GelMA/SilMA; Cryo-MNs	MMP-responsive; Local diffusion	Caspase-8/GSDME axis	Mode-Switch: Shifts pyroptosis back to silent apoptosis; clears pannus	(11, 68)
Bio-Catalytic & Sonodynamic Tools	D.ZAN (ZnO); MFO Nanozymes; H2-nanogen	Ultrasound; Redox-sensing; Piezocatalysis	NLRP3/GSDMD/GPX4	Microenvironment Reset: Targeted FLS pyroptosis or ferroptosis; scavenges systemic ROS	(33, 65)
Stimuli-Responsive Nanocarriers	CLT-FELipos; S-G Nanohydrogel	Enzyme-triggered; NF-B/MAPK cues	ER Stress/FAP+ FLS	Selective Lethality: Eradicates aggressive FLS while sparing healthy chondrocytes	(63, 70)
Biomimetic & Vesicular Relays	D@ApoEVFasL; Erythrocyte-membrane	Cell-cell recognition; Hepatic APC targeting	MerTK/Fas pathway	Resolution Reset: Restores efferocytosis; establishes antigen-specific tolerance	(62, 79)

### 5.4 Translational roadmap: challenges and mitigation

i. Off-target toxicity: Systemic nanoparticle exposure risks hepatorenal accumulation. Mitigation strategies include:

Tissue-specific targeting: Conjugating collagen II-binding peptides or folate receptor-α ligands to target activated FLS (62).

Dual-gating release: Requiring both inflammatory cues (ROS, acidic pH) and external triggers (e.g., focused ultrasound) to prevent premature cargo release.

Biodegradable scaffolds: Utilizing FDA-approved polymers (PLGA, chitosan) to streamline regulatory approval.

ii. Patient stratification: We propose a three-tier biomarker algorithm to identify responders:

Tier 1 (Imaging): [^11^C]CMP1 PET positivity identifies RIPK1+ “hotspots” suitable for death-mode interventions.

Tier 2 (Molecular): Circulating m6A-modified NLRP3/GSDMD transcripts (liquid biopsy) (9) confirm active PANoptotic signaling.

Tier 3 (Clinical): Relay profiling distinguishes seropositive cases (lung-joint axis markers) from seronegative cases (gut-metabolic signatures), as detailed in Section 4.4.

iii. Regulatory and Manufacturing: Clinical translation requires overcoming batch-to-batch variability via microfluidic synthesis for GMP compliance, and mitigating immunogenicity through PEGylation or zwitterionic coatings.

## 6 Summing up and looking forward

The paradigm of rheumatoid arthritis (RA) management is transitioning toward “Precision PANoptics,” shifting from broad cytokine suppression to the targeted “editing” of cellular death scripts. This Review has delineated a systemic framework where the arthritogenic synovial niche is governed by an integrated interplay between an epitranscriptomic “software” layer (m6A modification) and a molecular “hardware” scaffold (the PANoptosome), both of which are primed by multi-organ relays across the lung–joint, gut–joint, and neuro-immune axes.

Critical translational gaps must be acknowledged: Current mechanistic insights—particularly the USP5-METTL14-GLUT1 “glycolytic shield” (38) and Caspase-8 substrate switch (11)—derive primarily from cell lines and murine CIA models, with limitations including species-specific differences, incomplete recapitulation of human D2T-RA chronicity, and absence of validation in human synovial tissue using spatial metabolomics. Moreover, spatiotemporal heterogeneity of PANoptosis across RA subtypes remains unexplored. The differential engagement of systemic relays—lung–joint axis in seropositive RA (ACPA/ionic flux) (12, 23, 24) versus gut-metabolic/mechanical triggers in seronegative disease (palmitic acid/Piezo1) (29, 50)—suggests distinct PANoptotic kinetics requiring subtype-stratified spatial profiling across synovial compartments and disease stages. Furthermore, translating “death-mode editing” into practice faces specific hurdles: off-target nanotherapy toxicity requires tissue-specific targeting strategies (synovial homing peptides, dual-gating release; Section 5.4); patient stratification demands integrating [^11^C]CMP1 PET, m6A liquid biopsies, and relay biomarkers into a three-tier algorithm; and regulatory approval necessitates GMP-compliant manufacturing and immunogenicity mitigation.

To translate these insights into clinical reality via a phased roadmap, future research must: (1) leverage spatial transcriptomics and [^11^C]CMP1 PET imaging to map the spatiotemporal heterogeneity of the “synovial mosaic” in patients; (2) initiate Phase I safety trials of tissue-targeted nanotherapies with dose escalation and pharmacokinetic profiling; (3) establish multi-center biomarker validation studies correlating PET/m6A signatures with treatment response to enable Phase II stratification; and (4) bridge the neuro-efferocytic gap in the dorsal root ganglia (DRG) to resolve persistent pain independent of local inflammation. Bio-responsive nanoplatforms capable of switching Caspase-8 substrates from lytic GSDME cleavage back to silent apoptosis will enable the fundamental reprogramming of the arthritogenic microenvironment. Ultimately, by resetting the software of resolution and repairing the hardware of death, the field moves toward antigen-specific, drug-free remission—offering the potential for a definitive cure by restoring synovial death-mode homeostasis (80).
